# Dawn of the dialogue: AI's leap from lab to living room

**DOI:** 10.3389/frai.2024.1308156

**Published:** 2024-03-04

**Authors:** Tyler Thomas Procko, Timothy Elvira, Omar Ochoa

**Affiliations:** Department of Electrical Engineering and Computer Science, Embry-Riddle Aeronautical University, Daytona Beach, FL, United States

**Keywords:** Artificial Intelligence, Large Language Models, generative AI, ChatGPT, AI evolution

## Abstract

Prior to the advent of mainstream Large Language Models, e.g., ChatGPT, there were two contexts of AI use: theoretical and technical. The former involves the mathematics behind AI constructs, as well as new AI research; the latter encompasses the substance of AI use, i.e., programming, training, execution, etc. With the recent proliferation of Large Language Models for content generation, such as texts, images, and videos, there arises a new context of AI use: practical. This aspect of AI use is unique, in that practical users do not need theoretical or technical AI knowledge to prosper: they need only know how to prompt. In effect, the practical context of AI use is a black-box approach. These three contexts of AI converge in a unique intersection of AI knowledge. This emerging AI perspective is important to consider, as most AI users, now and in the future, will possess no deep knowledge of AI.

## 1 Introduction

The dawn of Artificial Intelligence (AI) was in 1950, with Alan Turing, who proposed the question “Can machines think?” and defined the imitation game, i.e., the Turing Test, which measures a machine's ability to exhibit intelligent behavior indistinguishable from that of a human (Turing, 2009). In the same year, a proposed application of AI was for a machine to play the game of chess (Shannon, 1950). Such nascent work led to the first neural network perceptron being implemented in 1957 and large expert systems being used in the 1980s (Lu, 2019). The AI winter of the 1980s occurred because of the limitations of such large expert systems, which are very rigid and require manual updates (Hendler, 2008).

Beginning in the early 2000s, adaptive Machine Learning (ML) models, including neural network models, were accelerating in many areas of interest, including object detection, recommendation systems, and Natural Language Processing (NLP), which focuses on the interaction between computers and humans through the medium of natural language. A Language Model (LM) is a statistical model that predicts the next word in a sequence of words. LMs are used for many tasks, e.g., text generation, speech and handwriting recognition, and machine translation.

In 2007, Google released a technique for training LMs on up to 2-trillion pieces of text, to produce Large Language Models (LLMs) remembering of up to 300-billion word combinations (Thorsten et al., 2007). The advancement of neural networks and particular activation functions in combination with expandingly powerful Graphics Processing Units (GPUs) in computers, allowed neural networks to become *deeper*, incorporating more layers and complex architectures (Wang and Raj, 2017; Alom et al., 2018; Jeffrey, 2022). This progress engendered the current trend of Deep Learning (DL) which is at the heart of the rapid expansion and adoption of modern LLMs in many areas of life.

In 2017, a groundbreaking neural network architecture, the Transformer, was proposed (Vaswani et al., 2017). Built upon the Transformer, OpenAI's Generative Pre-trained Transformer (GPT) series is the flagship LLM of the modern world (Radford et al., 2018, 2019; Brown et al., 2020). From its web interface, GPT allows anyone, layman or ML expert, to “chat” with an LLM trained on billions of texts in natural language, deriving insight and knowledge. GPT is used frequently by software developers to write and test their code, by digital content creators to assist in creative tasks, by students to help with homework, by educators to create course material, and all manner of scenarios, professional or personal (Zhang et al., 2023). Similarly, LLMs power approaches such as DALL-E (Ramesh et al., 2022) and stable diffusion (Rombach et al., 2022), that allow users to generate images from text prompts.

Before LLMs (principally, OpenAI's GPT) emerged, there were two distinct domains of expertise, i.e., usage contexts, of ML: *theoretical* and *technical*. The former is embodied in research papers and the mathematics behind ML models by researchers; the latter is expressed in the training, programming, testing, and use of ML models by ML engineers. Now, with large-scale generative AI constructs like GPT having become mainstream objects of worldwide fascination, a third context of ML use has arisen: *practical*. The implications of this new ML context are important to consider for the future of ML.

## 2 A changing landscape

**Figure 1:**
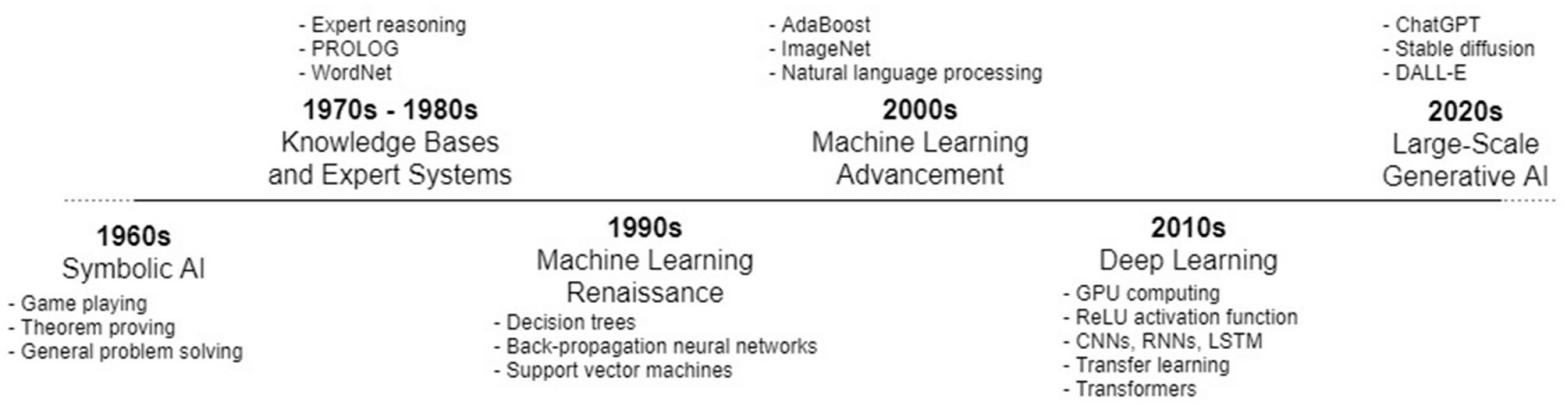
A basic chronology of the evolution of AI trends (note: some constructs, e.g., CNNs and RNNs, were proposed as early as the 1980s, but only gained popularity later in the 2010s).

A topographic view of AI use since its inception would reveal that AI was historically created for specific purposes, e.g., optimization problems, data analysis and so on (Lu, 2019). See Figure 1. Moreover, AI use was primarily relegated to the researchers, scientists, and engineers working with, and producing, such constructs. Uninformed users of various applications may have encountered the result of some AI module, e.g., as Web search engines have applied AI to rank search results for decades; but these interactions were beneath the surface of the application interfaces, i.e., users did not explicitly interact with the underlying AI.

Only recently has the possibility of general AI become feasible outside of AI research, and easily accessible by the public, where non-AI experts can interact, in natural language, with generally intelligent agents capable of responding dynamically to any given prompt. This point in humanity's technological progress is unprecedented, as interaction with such agents allows users to derive instant, effortless insight, assistance in self-reflection, knowledge synthesis, and any number of other cognitive tasks.

In 1960, J. C. R. Licklider, the grandfather of the World Wide Web and one of the founding fathers of the ARPANet project, outlined the future of machine intelligence, asserting that the new age of computing would see what he termed “man-computer symbiosis” (Licklider, 1960). A large 2019 literature review on the trend of AI over time concluded with the following: “… AI will tend to be more extensive and generalized… The emergence of intelligent systems means the innovation of traditional mechanisms, from human to machine collaboration” (Lu, 2019). As of around 2020, large-scale generative AI constructs from OpenAI, e.g., GPT-3, GPT-3.5, and GPT-4, have effectively made man-computer symbiosis part of the normal course of life for most Internet users, underpinning a societal “megatrend” with broad and massive impacts on the world (Haluza and Jungwirth, 2023).

**Figure 2:**
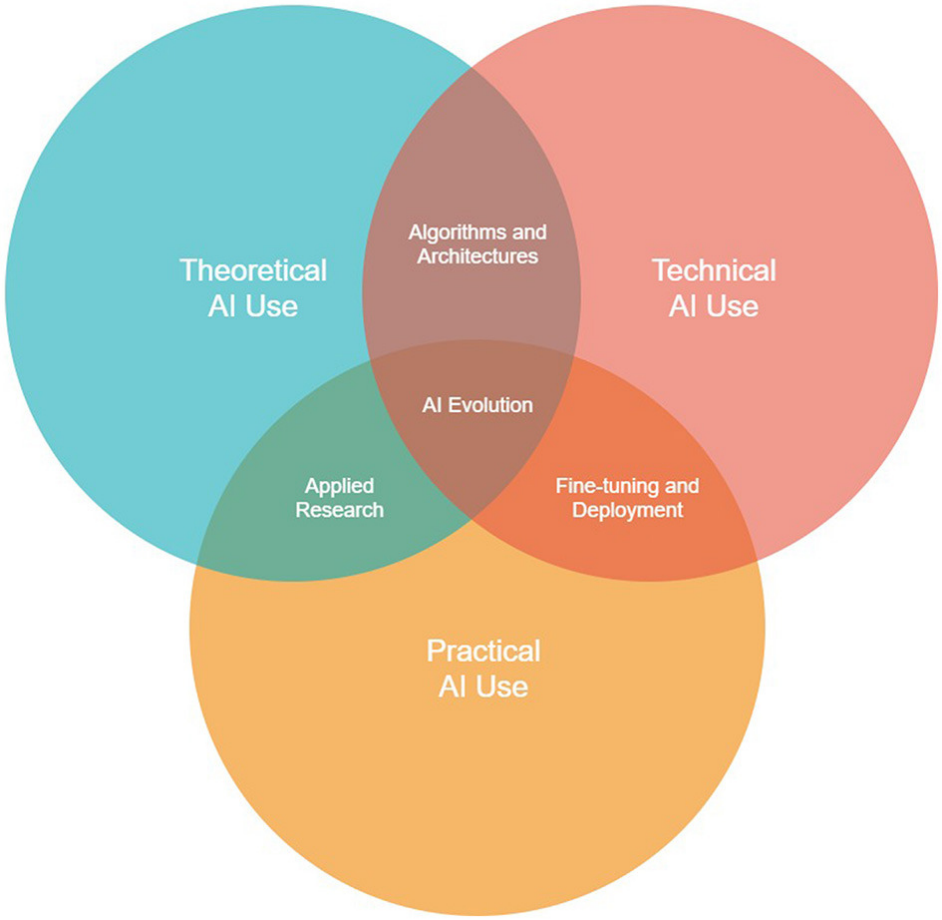
The three intersecting knowledge contexts of AI use: theoretical, technical, and practical.

Already these earlier predictions are perfectly salient. A report published in association with OpenAI found that, in the coming years, 80% of American workers may have at least 10% of their tasks affected by LLMs, and 19% of workers may have at least 50% of their tasks affected (Eloundou et al., 2023). Some have stated that, by 2025, over 90% of online content will be generated by AI (not by AI, 2023). It is now clear that the two traditional contexts of AI use, i.e., *theoretical*, and *technical*, have been joined by another, *practical*, in which non-AI experts interact with AI constructs in their own natural language, having no real understanding of the underlying theory or technical elements (see Figure 2).

- *Theoretical AI use*—The context encompassing AI research and theory, particularly involving the mathematics underlying AI constructs, e.g., neural network activation functions, language model matrix calculations, etc.
- *Technical AI use*—The context within which AI engineers program, debug, train, and test AI constructs, and integrate them with larger software systems.
- *Practical AI use*—The skill of strategically navigating the volatility and nuances (the “personalities”) of generative AI constructs through “black-box” means, e.g., prompting, learning trends, etc.; i.e., the “layman” approach to AI.

## 3 Practical AI knowledge

The use of AI has clearly shifted toward mass use for content-generative purposes, i.e., the *practical context*. The rapid proliferation of this context has implications of primary concern to both AI developers and users. The consequences of capable general LLMs, such as ChatGPT, being available to the average individual, are given below:

- *Individuals will refer to them first*—LLMs are effortless to access and can provide insight on many topics, more than even face-to-face discussion with a multitude of human experts can; as such, individuals will use them to consult on various topics before seeking human assistance.
- *They will learn from us*—Reinforcement Learning from Human Feedback (RLHF) (Ouyang et al., 2022) has been and will be used to fine-tune LLMs to excel in particular domain areas, reduce noisy output and adhere to policies, e.g., business or governmental logic.
- *They will be as good as what they are given*—The limitations of LLMs will be exhibited because of their training data; future expansion of LLMs will likely center around the inclusion of new online data sources and manual training data sanitization.
- *Online content will be increasingly AI-generated*—As LLMs and other generative AI gain popularity, digital and online content will more frequently be generated with AI (not by AI, 2023).
- *Intellectual property (IP) laws will change*—As more digital content will be AI-generated, the question of “who owns it?” will arise; already, there are discussions of reforming IP statutes, e.g., for digital media and patented inventions, in the face of large-scale generative AI (Ramalho, 2018; Abbott and Rothman, 2022).
- *Education will change*—Students consistently use ChatGPT to help with homework: to some, this may be considered cheating (Procko et al., 2023); also, some educators use ChatGPT to prepare lecture materials, raising questions about the current state and future of education, with some wondering if it should be banned from academia altogether (Yu, 2013).
- *Job skills will change*—Computer-oriented or otherwise non-physical jobs will be impacted by large-scale generative AI (Eloundou et al., 2023): many will benefit from the generalizable consulting capabilities of LLMs, many will require explicit expertise in prompting LLMs, while some may wholly be replaced in the future, leaving humans mostly “dumb” jobs (Trajtenberg, 2018).
- *AI-generated content may result in negative training feedback loops*—As online content will be generated primarily by AI from any number of sources, it is possible that training future AI will encounter issues of training on synthetic data if not appropriately labeled as such.

Also likely to occur is growing competition between OpenAI (which, until now, has essentially possessed a monopoly on large-scale generative AI) and third parties, resulting in innovation and further improvements to the generalizability of LLMs. In effect, the availability of powerful LLMs such as ChatGPT for the general public has elevated general AI out of the minds of researchers and codebases of developers, and into the daily routines of the great masses of humanity, engendering worldwide, emphatic interest, a source of essentially unlimited improvement [refer to OpenAI's GPT-4 technical report which discusses RLHF (OpenAI, 2023)], and a burgeoning, competitive financial sector in its own right.

The feasibility of Artificial General Intelligence (AGI) has long been questioned. It seems that only in recent years, beginning, for most, with the mainstream adoption of ChatGPT, AGI is no longer a prognostication of tomorrow, but a possibility of today. Interestingly, in a 2018 socioeconomic study, AI was proposed to be the next General Purpose Technology (GPT) (not to be confused with the 2020s term “GPT”, i.e., Generative Pre-trained Transformer); the study states “… many occupations will indeed vanish with the advent of AI as the new General Purpose Technology…” (Trajtenberg, 2018). Some of the job or job areas likely to be replaced by large-scale generative AI are: stock trader, bookkeeper, accountant, data analyst, human resources, paralegal, research assistant, proofreader, copywriter, content writer, online tutor, social media manager, receptionist, customer service, data entry, translator, editor, journalist, and graphic designer, among many others (Singh, 2023).

## 4 A call for consensus

Given the implications of large-scale generative AI reaching a position of universal interest with the public, and the forthcoming impacts to online content, intellectual property, occupations, and education, there must be established a consensus among persons regarding the limits of large-scale generative AI use.

It is necessary to carefully delineate between Human-Enhancing Innovations (HEI), e.g., human sensory enhancement, and Human-Replacing Innovations (HRI) (Trajtenberg, 2018). In many ways, LLMs such as ChatGPT are HEIs, insofar as they are used iteratively to assist humans in various insight gleaning tasks that would otherwise take much longer; yet LLMs can, and likely will be, utilized as HRIs in certain occupational areas (Singh, 2023). There is, figuratively speaking, a stark line drawn in the socio-ethical sand, on the other side of which rest application areas that AI has not touched (and, some may argue, *should never* touch), e.g., surgical practice. Notwithstanding these sentiments, humans must make the most of these technologies, and “search for ways to ameliorate the detrimental effects of AI, and enhance its positive ones” (Trajtenberg, 2018).

Channeling the insights of J. C. R. Licklider in the 1960s, it is incumbent on us to fully actualize both our human potential, and the potential of large-scale generative AI, effectively dictating our terms for the coming age of man-computer symbiosis, an age in which mundane tasks can be performed by generalized agents, ameliorating common impedances for a great deal of undertakings and allowing man to progress with less friction.

As such, the appropriate response is to embrace the intimate integration of large-scale generative AI with human operations, by reconciling it with both educational and ethical systems. First, AI knowledge should, ideally, be included at all levels of education, akin to history or government studies in school curricula, to prepare younger generations for an AI-enabled world and changing job skills; moreover, educators must alter student assessment to account for the ubiquitous nature of AI (Trajtenberg, 2018; Procko et al., 2023). Second, ethical statutes, e.g., those pertaining to intellectual property, may have to evolve to include authorship or ownership based on the practical use of large-scale generative AI, e.g., through prompting (Abbott and Rothman, 2022). A recommendation sensible for the digital arena is to include a “not by AI” image badge on all content (e.g., art, text, music, etc.) generated primarily by humans. This is important, not only to separate AI- and human-generated content, but to broadcast safety from the unpredictable outputs of AI (not by AI, 2023).

## 5 Discussion

The use of AI has shifted dramatically considering recent developments, most notably, the availability of large-scale generative AI to the public. This shift has transformed the most common domain expertise of AI from the theoretical-technical context, into the practical, or “layman”, context, where non-AI experts derive insight from generative AI constructs, e.g., ChatGPT, by simply prompting in their natural language. This shift has implications for individual thought processing, digital media, intellectual property laws, academia, and job skills. In some occupation areas, indeed, humans may be replaced, or have their work significantly augmented, by the new wave of Large Language Models. As amelioration, education curricula should include AI knowledge for students young and old, to meet changing job demands; and, intellectual property laws will have to change to retain authorship incentive for created works.

The coming age of computer interaction will invariably see humans and AI grow together, in the ultimate direction of a hybrid, augmented intelligence. Humans must be cognizant of the significant impacts of large-scale generative AI, and act to delineate its limits in interaction with human operations, while understanding that such constructs provide the opportunity for man-computer symbiosis, or world-scale collaboration between man and machine, facilitating faster thought, learning, research, and progress in many application areas.

## Data availability statement

The original contributions presented in the study are included in the article/supplementary material, further inquiries can be directed to the corresponding author.

## Author contributions

TP: Visualization, Writing – original draft, Writing – review & editing. TE: Conceptualization, Writing – review & editing. OO: Writing – review & editing.
